# Supplementary material for: Variational autoencoder-based chemical latent space for large molecular structures with 3D complexity
Source: Commun Chem. 2023 Nov 16;6:249. doi: 10.1038/s42004-023-01054-6 (PMC10654724; doi:10.1038/s42004-023-01054-6)

Supplementary Materials:

"Variational autoencoder-based chemical latent space for large molecular structures with 3D complexity"

Toshiki Ochiai, Tensei Inukai, Manato Akiyama, Kairi Furui, Masahito Ohue, Nobuaki Matsumori, Shinsuke Inuki, Motonari Uesugi, Toshiaki Sunazuka, Kazuya Kikuchi, Hideaki Kakeya, and Yasubumi Sakakibara

Supplemental Methods

1. NP-VAE algorithm

The preprocessing of NP-VAE

There are two objectives in the preprocessing of NP-VAE. The first one is to convert the input compound structure into a simpler structure that can be more easily handled. Particularly when dealing with large molecular structures, calculating at the single-atom level would result in an enormous order both in time and space. To address this, we devised a procedure to capture compound structures by decomposing them into several fragments (substructures). Also, the presence of loop structures in the molecular graph would require a significant computational cost during compound generation in the subsequent Decoder; thus, we aim to capture the structure as a tree without loops. The second objective is to reshape the compounds so that meaningful physicochemical features can be extracted. Aromatic rings like benzene, as well as functional groups deeply involved in the physicochemical properties, such as amide and carboxyl groups, should be treated as a single fragment rather than a sequence of individual atoms. The compound decomposition algorithm was determined based on these objectives.

In the preprocessing step, we first extract substructures fragmented from the entire compound structures according to the following decomposition procedure, and save them as substructure labels while converting them into corresponding tree structures (Supplementary Figure S3).

[Decomposition of compound structures into substructures (fragment units)]

1. Define the compound structure as $G = (V, E)$ (where V, E represent atoms and bonds, respectively), and let the adjacent two atoms be v_i, $v_j (\in V)$, and their bond be $e_{ij} (\in E)$. When e_{ij} is not a ring bond, and both or either of v_i, v_j are in a ring structure, and both have a bond order greater than 1 excluding hydrogen atoms, remove e_{ij} and decompose. This yields substructures $\mathcal{G}_1$, $\mathcal{G}_2$,…, $\mathcal{G}_N$.
2. For each of the substructures $\mathcal{G}_1$, $\mathcal{G}_2$,…, $\mathcal{G}_N$, select those that do not have a ring structure and have a usage frequency of less than f_c times in the dataset. Denote

these as $\mathcal{G}'_1$, $\mathcal{G}'_2$,…, $\mathcal{G}'_n$. The ones not selected in this step are saved as substructure labels.

3. Perform functional group-focused decomposition for each of the substructures $\mathcal{G}'_1$, $\mathcal{G}'_2$,…, $\mathcal{G}'_n$. First, the targeted functional group is an amide group or amide bond. Define the substructure as $\mathcal{G}'_m = (\mathcal{V}_m, \mathcal{E}_m)(1 \leq m \leq n)$ and let the adjacent two atoms be v_i, $v_j(\in \mathcal{V}_m)$ and their bond $e_{ij}(\in \mathcal{E}_m)$. If either v_i or v_j is within the amide C(=O)N, and the other is not a hydrogen atom, remove their bond e_{ij} and decompose. Save C(=O)N as a substructure label, and denote the other substructures as g^1_1, g^1_2,…, $g^1_{n'}$.
4. Perform a carboxyl group or ester bond-focused decomposition for each of the substructures g^1_1 , g^1_2 ,…, $g^1_{n'}$. Define the substructure as $g^1_m = (v^1_m, e^1_m)(1 \leq m \leq n')$ and let the adjacent two atoms be v_i, $v_j(\in v^1_m)$ and their bond $e_{ij}(\in e^1_m)$. If either v_i or v_j is within the carboxyl or ester bond C(=O)O, and the other is not a hydrogen atom, remove their bond e_{ij} and decompose. Save C(=O)N as a substructure label, and denote the other substructures as g^2_1, g^2_2,…, $g^2_{n''}$.
5. Perform an aldehyde or ketone-focused decomposition for each of the substructures g^2_1, g^2_2,…, $g^2_{n''}$. Define the substructure as $g^2_m = (v^2_m, e^2_m)(1 \leq m \leq n'')$ and let the adjacent two atoms be v_i or v_j and their bond $e_{ij}(\in e^3_m)$. If either v_i or v_j is in the aldehyde or ketone group C(=O) and the other is not a hydrogen atom, remove their bond e_{ij} and decompose. Save C(=O) as a substructure label and define the remaining substructures as g^3_1, g^3_2,…, $g^3_{n'''}$.
6. Lastly, for each of the substructures g^3_1 , g^3_2 ,…, $g^3_{n'''}$, decomposition is performed focusing on hydroxyl groups or ether bonds. Define the substructure as $g^3_m = (v^3_m, e^3_m)(1 \leq m \leq n''')$ and let the two adjacent atoms be v_i, $v_j(\in v^3_m)$ and their bond $e_{ij}(\in e^3_m)$. If either v_i or v_j is an oxygen atom and the other is a carbon atom, remove their bond e_{ij} and decompose. Save all the resulting substructures as substructure labels.

When defining the tree structure $\mathcal{T}$ corresponding to the compound structure G, the number of nodes in $\mathcal{T}$ matches the number of substructures, and edges are drawn between neighboring substructures within G. At each node of $\mathcal{T}$, the ECFP calculated from the corresponding substructure is stored as a feature vector.

The root node of each tree structure is determined based on the CANGEN algorithm of RDKit [25]. In other words, the priority of each atom is determined based on its connectivity value and atomic number, and the root node is uniquely determined by this priority.

NP-VAE encoder

In the Encoder, feature extraction of compound structures is performed combining two processes (Supplementary Figure S4). First, for each ECFP stored in the nodes of the tree structure $\mathcal{T}$, a feature vector h is obtained using Child-Sum Tree-LSTM [22]. Let $C(j)$ be all the child nodes of node j, x_j be the ECFP of node j, h_j be the hidden state of node j in the Tree-LSTM, i_j be the input gate, o_j be the output gate, c_j be the memory cell, and f_{jk} be the forget gate for child node k of node j. The Child-Sum Tree-LSTM is defined by the following equations:

$$h_j = o_j \odot \tanh(c_j)$$

$$o_j = \mathrm{sigmoid}\left(W^o x_j + U^o \tilde{h}_j + b^o\right)$$

$$\tilde{h}_j = \sum_{k \in C(j)} h_k$$

$$c_j = i_j \odot u_j + \sum_{k \in C(j)} f_{jk} \odot c_k$$

$$i_j = \mathrm{sigmoid}\left(W^i x_j + U^i \tilde{h}_j + b^i\right)$$

$$u_j = \tanh\left(W^u x_j + U^u \tilde{h}_j + b^u\right)$$

$$f_{jk} = \mathrm{sigmoid}\left(W^f x_j + U^f h_k + b^f\right)$$

$$\mathrm{sigmoid}(p) = \frac{1}{1 + \exp(-p)}$$

$$\tanh(p) = \frac{\exp(p) - \exp(-p)}{\exp(p) + \exp(-p)}$$

Here, $\odot$ represents the element-wise product, $W^i, W^f, W^o, W^u, U^i, U^f, U^o, U^u$ are the weights learned in the fully connected layers, and b^i, b^f, b^o, b^u are the learned constants (biases).

Second, we compute the ECFP for the entire compound structure. This is denoted as x_0, and by inputting it into the L-layer fully connected layers, we obtain the output x_L. The output x_L is defined by the following formula, with the weights and biases of the l-th fully connected layer denoted as W^l and b^l, respectively.

$$x_l = W^l x_{l-1} + b^l \quad (1 \leq l \leq L)$$

Lastly, we sum up the feature vector h_0, which corresponds to the root node obtained from the Tree-LSTM, and the output x_L of the fully connected layers. Using random noise $\varepsilon \sim N(0, I)$, we compute the latent variable z via the reparameterization trick. With the weights of the fully connected layers denoted as $W^{enc}, W^{\mu}, W^{\sigma}$ and biases as $b^{enc}, b^{\mu}, b^{\sigma}$, the expression is as follows.

$$z = \mu + \varepsilon \odot \sigma$$

$$\mu = W^{\mu} h_G + b^{\mu}$$

$$\sigma = W^{\sigma} h_G + b^{\sigma}$$

$$h_G = [W^{enc}(h_0 + x_L) + b^{enc}]$$

NP-VAE decoder

In the Decoder, based on the input latent variable z, a tree structure is generated using a depth-first sequential algorithm and is then converted to a compound structure for output (Supplementary Figure 5). NP-VAE decoder consists of seven procedures: Root label prediction, Topological prediction, Bond prediction, Label prediction, Update the variable z, Conversion to compound structure, and Chirality Assignment.

[Root label prediction]

In the first step of the Decoder, called Root label prediction, we predict the substructure label that will be assigned to the initially generated root node. The prediction of substructure labels is selected from all the substructure labels obtained during the preprocessing of NP-VAE. The input latent variable z to the Decoder is fed into L_r fully connected layers, and a multi-class classification is performed. The output u_{L_r} is

represented by the following equations, where W_r^l, b_r^l are the weights and biases of the fully connected layer at the l-th level, respectively:

$$u_l = \tanh(W_r^l u_{l-1} + b_r^l) \quad (1 \le l \le L_r - 1)$$

$$u_{L_r} = \mathrm{softmax}(W_r^{Lr} u_{L_r-1} + b_r^{Lr})$$

Here, $u_0 = z$, and the index of the selected substructure label is $\mathrm{argmax}(u_{L_r})$. Note that:

$$\mathrm{softmax}(p_i) = \frac{\exp(p_i)}{\sum_j \exp(p_j)}$$

[Topological prediction]

In Topological prediction, we predict whether or not to generate a new child node under the current node. If it is predicted to generate a child node, we then proceed to bond prediction and label prediction. On the other hand, if it is predicted not to generate a child node, we terminate the Decoder process (Break) if the node is at the root position; otherwise, we backtrack from the current node to its parent node (Backtrack). Input z_t is fed into the fully connected layer to perform binary classification. The output u_τ is represented by the following equation, where W_τ, b_τ are the weights and biases of the fully connected layer, respectively:

$$u_\tau = \mathrm{softmax}(W_\tau z_t + b_\tau)$$

When $\mathrm{argmax}(u_\tau) = 0$, no child node is created, and when $\mathrm{argmax}(u_\tau) = 1$, an attempt is made to generate a child node.

[Bond prediction]

In Bond prediction, we predict the type of bond between the current node's substructure and the substructure of the newly generated child node. Input z_t is fed into the L_b-layer fully connected layer to perform ternary classification. The output u_{L_b} is represented by the following equations, where W_b^l, b_b^l are the weights and biases of the l-th fully connected layer, respectively:

$$u_l = \tanh\left(W_b^l u_{l-1} + b_b^l\right) \quad (1 \le l \le L_b - 1)$$

$$u_{L_b} = \mathrm{softmax}(W_b^{L_b} u_{L_b-1} + b_b^{L_b})$$

Here, $u_0 = z_t$, and when $\mathrm{argmax}\left(u_{L_b}\right) = 0$, a single bond is attempted, when $\mathrm{argmax}\left(u_{L_b}\right) = 1$, a double bond is attempted, and when $\mathrm{argmax}\left(u_{L_b}\right) = 2$, a triple bond connection is attempted.

[Label prediction]

In Label prediction, we predict the substructure label that corresponds to the newly generated child node. Input z_t is fed into the L_s-layer fully connected layer to perform multiclass classification. The output u_{L_s} is represented by the following equations, where W_s^l, b_s^l are the weights and biases of the l-th fully connected layer, respectively:

$$u_l = \tanh(W_s^l u_{l-1} + b_s^l) \quad (1 \leq l \leq L_s - 1) \qquad (L1)$$

$$u_{L_s} = \mathrm{softmax}(W_s^{L_s} u_{L_s-1} + b_s^{L_s}) \qquad (L2)$$

Here, $u_0 = z_t$, and the index of the selected substructure label is $\mathrm{argmax}(u_{L_s})$.

If not in training mode, after Label Prediction, it is checked whether the selected substructure label can be connected to the parent node's substructure label (resulting in a chemically valid structure after connection). If the connection is successful, the next target node is set as the newly generated child node. If the connection cannot be made correctly, the prediction in Bond Prediction is changed from triple bond to double bond, or from double bond to single bond, and the connection is attempted again. If the connection still cannot be made with a single bond, the connection is attempted with the substructure label with the next highest prediction probability in equation ($L2$). If the connection still fails, the connection is repeatedly attempted with the higher predicted substructures up to the specified top *n*. If all connections fail, the generation of the child node is stopped, and the current node's parent node is set as the next target node (Backtrack).

[Update z]

After label prediction or backtrack, we compute z_{t+1} from z_t using a fully connected layer. The output z_{t+1} is defined by the following equation, where W and b are the weights and biases of the fully connected layer, respectively.

$$z_{t+1} = \tanh(W(z_t + h_i) + b)$$

Here, h_i is the feature vector obtained by performing the Child-Sum Tree-LSTM computation, which represents the features at node i after propagating the ECFP stored in each node in the tentative tree structure. During child node generation, the features are transmitted through backward propagation from the root node to the leaf node, and that child node is set as node i (Supplementary Figure S6(a)). On the other hand, during backtrack, after the backward propagation from the root node to the leaf node, a forward

propagation from the leaf node to the root node is performed, and the Backtrack destination parent node is set as node i (Supplementary Figure S6(b)).

[Conversion to the corresponding compound structure]

In Conversion to compound structure, after generating the tree structure, the substructure labels of each node are connected and converted into the corresponding compound structure. Since information about the atoms corresponding to the bonding sites within the substructure and their bonding order is already included in the substructure labels, the compound structure can be uniquely determined from the generated tree structure (Supplementary Figure S6(c)).

[Chirality assignment]

In Assignment of chirality, to handle three-dimensional information of compounds in the Encoder, ECFP with chirality information is used. In the Decoder, the latent variable z is input to the L_c-layer fully connected layer, and the predicted ECFP value is output. The output u_{L_c} is defined by the following equation, where the weights and biases of the l-th fully connected layer are W_c^l and b_c^l, respectively.

$$u_l = \tanh(W_c^l u_{l-1} + b_c^l) \quad (1 \leq l \leq L_c - 1)$$

$$u_{L_c} = \mathrm{sigmoid}\left(W_c^{L_c} u_{L_c-1} + b_c^{L_c}\right)$$

$$u_0 = z$$

Here, the dimension of u_{L_c} is same as the bit size of ECFP. After the two-dimensional structure of the compound is output based on the aforementioned sequential algorithm, all possible stereoisomers are enumerated and their ECFP is calculated. The Euclidean distance between them and u_{L_c} is computed, and the three-dimensional structure corresponding to the ECFP with the smallest distance is selected as the output compound structure.

Learning

During training, to ensure proper learning, even if an incorrect prediction is made in the decoding process that cannot reconstruct the input data, the learning proceeds by propagating feature values on the tree structure, replacing it with the correct one for reconstruction. Additionally, to ensure that the latent space generated by NP-VAE not only accounts for structural information of compounds but also incorporates functional

information, such as bioactivity, the latent variable z is input to the L_p-layer fully connected layer for predicting the activity value of the input compounds. The output u_{L_p} is defined by the following formula, with the weights and biases of the l-th fully connected layer represented by W_p^l and b_p^l, respectively.

$$u_l = W_p^l u_{l-1} + b_p^l \quad (1 \le l \le L_p)$$

$$u_0 = z$$

By adding the difference loss between the predicted value u_{L_p} and the true activity value in the loss function, functional information is incorporated into the chemical latent space. The loss function during NP-VAE training consists of a weighted sum of the cross-entropy loss (CE) calculated from each prediction task in the Decoder, the KL divergence (D_{KL}) representing the distance between the distribution $Q(z|X)$ of latent variables and the Gaussian distribution, the binary cross-entropy loss (BCE) in three-dimensional structure prediction, and the mean squared error (MSE) in functional information prediction. Let the ground truth values for Root Label prediction, Topological prediction, Label prediction, and Bond prediction be y_r, y_τ, y_s, and y_b respectively (represented by a vector where the index of the correct label is 1 and all others are 0), and let the true ECFP value be y_c and the true functional information be y_p. The loss function L is defined as follows:

$$L = \alpha \cdot CE(y_r, u_{L_r}) + \beta \cdot \sum_i CE(y_{\tau,i}, u_\tau) + \gamma \cdot \sum_j CE(y_{s,j}, u_{L_s}) + \delta \cdot \sum_j CE(y_{b,j}, u_{L_b})$$
$$+\varepsilon \cdot BCE(y_c, u_{L_c}) + \epsilon \cdot MSE\left(y_p, u_{L_p}\right) + \zeta \cdot D_{KL}[Q(z|X) \| P(z)]$$

$$CE(y, \hat{y}) = -y\log\hat{y}$$

$$BCE(y, \hat{y}) = -[y\log\hat{y} + (1 - y)\log(1 - \hat{y})]$$

$$MSE(y, \hat{y}) = (y - \hat{y})^2$$

$$D_{KL}[Q(z|X) \| P(z)] = -\frac{1}{2}\sum_d (1 + \log\sigma_d{}^2 - \mu_d{}^2 - \sigma_d{}^2)$$

Here, α, β, γ, δ, ε, ϵ, and ζ are hyperparameters used to adjust the contribution of each term.

Supplemental Figures

Supplemental Figure S1. Compound structures for each dataset. Compared to the compounds in ZINC used in previous studies, the evaluation dataset contains compounds with a larger number of atoms. DrugBank and Project datasets are even larger and contain complex structures without repeating units.

Supplemental Figure S2. Plot illustrating the size distributions of the molecules generated by all models. Each distribution is highly divergent, indicating the generation of diverse molecular weights..

Supplemental Figure S3. Plot depicting the correlation between NP-likeness score and embedding distance. We calculated the correlation between the embedding distance and the difference of NP-likeness scores for randomly sampled pairs of points in the latent space.

Supplemental Figure S4. Frequency graph of NP-likeness scores for DrugBank, chemotherapy drugs, and molecular-targeted drugs. Compared to DrugBank overall, molecular-targeted drugs have a sharp peak at a lower position, while chemotherapy drugs have a peak at a higher position. It can be seen that the majority of molecular-targeted drugs have NP-likeness scores distributed within a limited range.

Supplemental Figure S5. Preprocessing of NP-VAE. (a) Decompose the compound structure by focusing on the ring structures and extract the resulting fragments as substructure labels. For linear substructures, if their usage frequency in the dataset is lower than the hyperparameter f_c, further decomposition focusing on functional groups and

bonds is performed to extract substructure labels. (b) Convert the compound structure to the corresponding tree structure. The number of nodes in the tree structure is equal to the number of substructures, with edges drawn between neighboring substructures. Each node stores the ECFP calculated from the corresponding substructure.

Supplemental Figure S6. Encoder architecture of NP-VAE. The encoder extracts compound structure features through two pathways. For the tree structure created in the preprocessing, Tree-LSTM is used to propagate the features from leaf nodes to the root node, obtaining the feature vector h_0 with micro-scale information. In addition, ECFP is calculated from the entire compound structure, and a feature vector x_L with macro-scale information is obtained through L layers of fully connected layers. Finally, the sum of the two feature vectors h_0 and x_L is input to a fully connected layer, and the latent variable z is calculated using the reparameterization trick.

Supplemental Figure S7. Decoder algorithm of NP-VAE. Based on the latent variable z, a compound structure is generated using a depth-first algorithm. In Root label prediction, the substructure corresponding to the root node is predicted. In Topological prediction, it is predicted whether to create a new child node under the current target node. In Bond prediction and Label prediction, the type of bond with the child node and the substructure corresponding to the child node are predicted, respectively. The latent variable z is sequentially updated and used for prediction in the next iteration. The obtained tree structure can be uniquely converted to a compound structure.

Supplemental Figure S8. Updating z. (a) If a child node is generated under the current target node, z is updated by reverse feature propagation from the root node to the leaf node in the provisional tree structure. (b) If a child node is not generated under the current target node and Backtracking is performed, z is updated by performing reverse feature

propagation in the provisional tree structure, followed by forward feature propagation in the opposite direction. (c) The substructure labels also store information about which atoms are bonded to adjacent substructures in which order. Therefore, substructures are uniquely bonded depending on the position of the target node, and the structure is converted from a tree structure to a compound structure.

Supplementary Figure S1

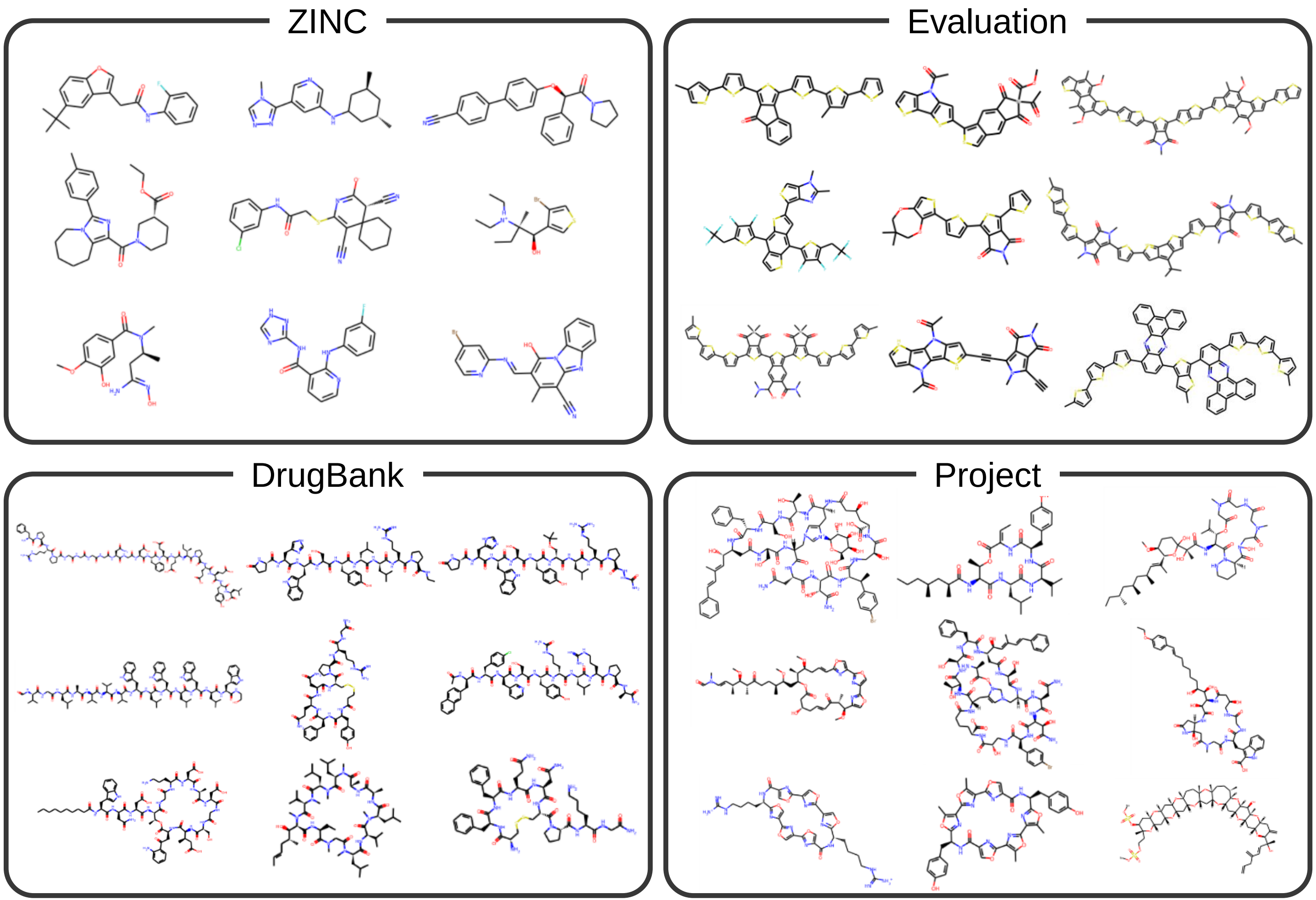

Supplementary Figure S2

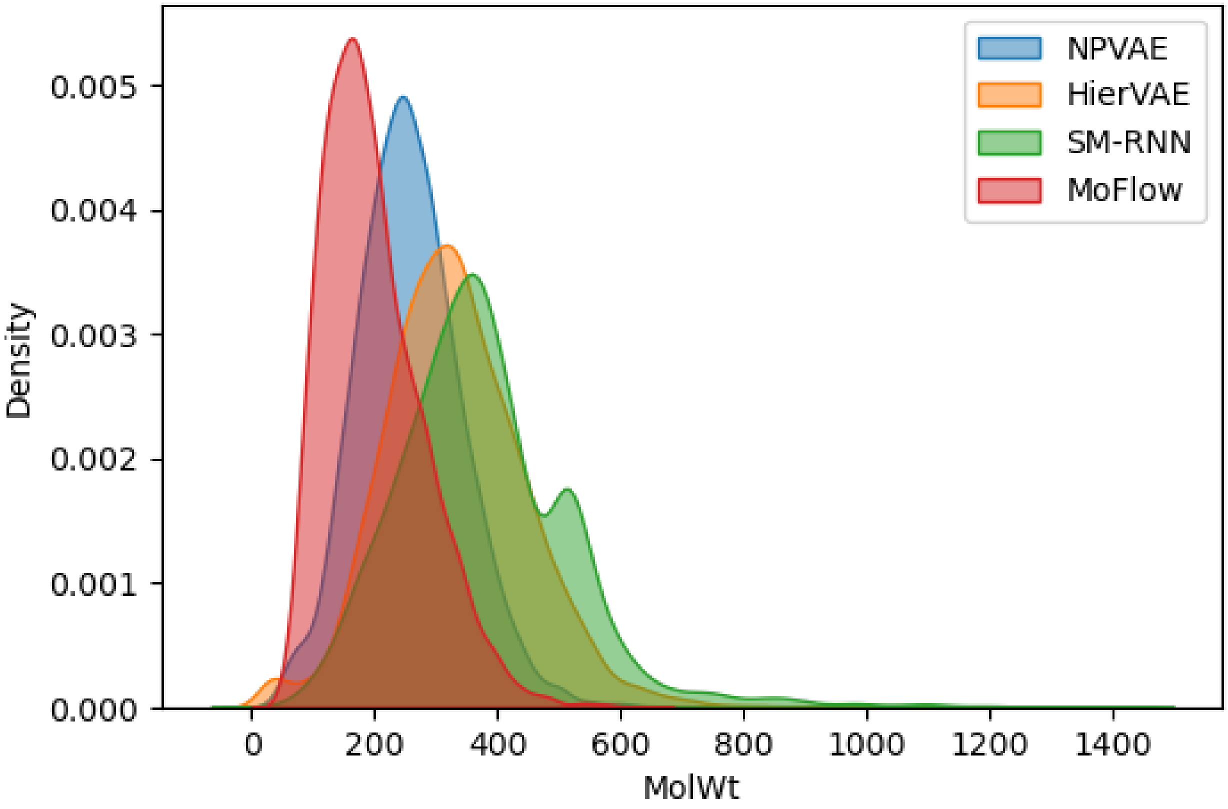

Supplementary Figure S3

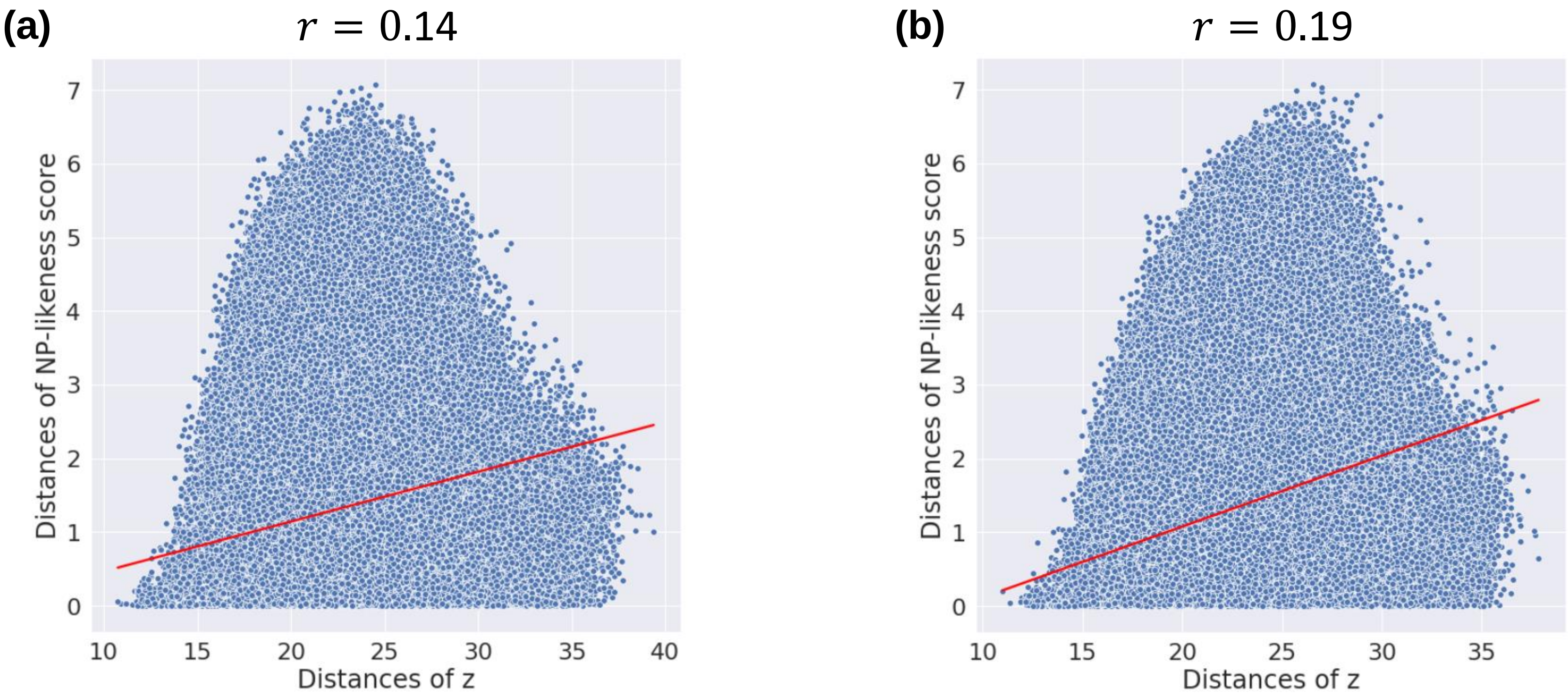

Pearson correlation between the embedding distance and the difference of NP-likeness scores for randomly sampled pairs of points, (a) in the latent space constructed by incorporating NP-likeness scores, and (b) in the latent space constructed using only the structural information.

Supplementary Figure S4

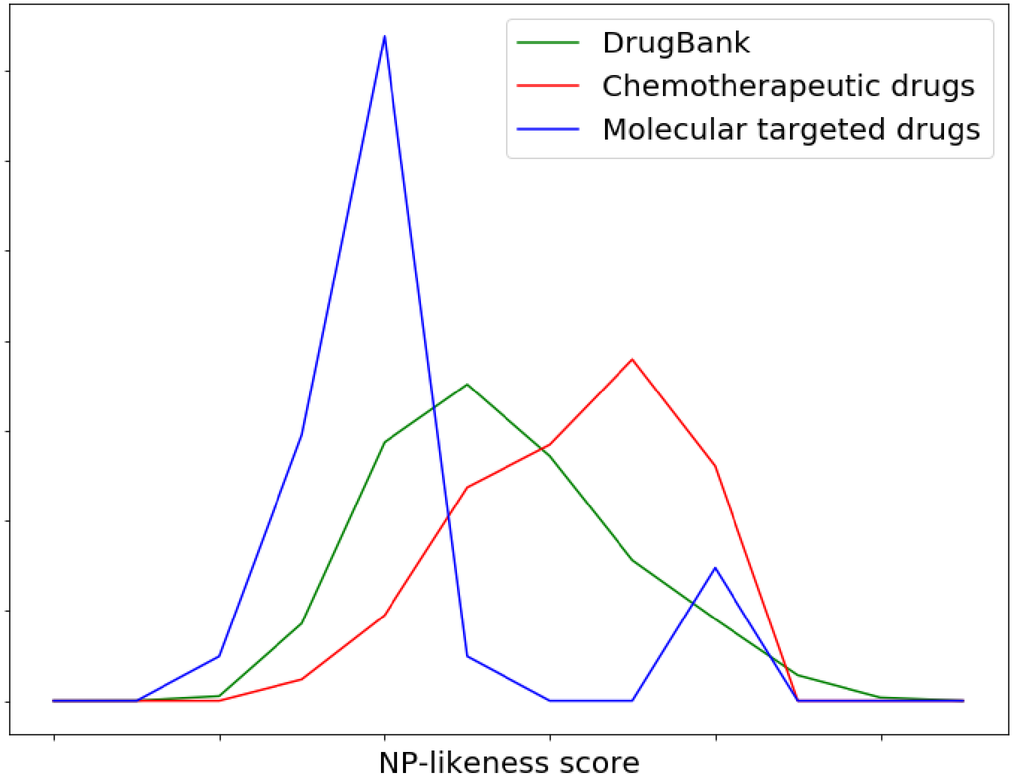

Supplementary Figure S5

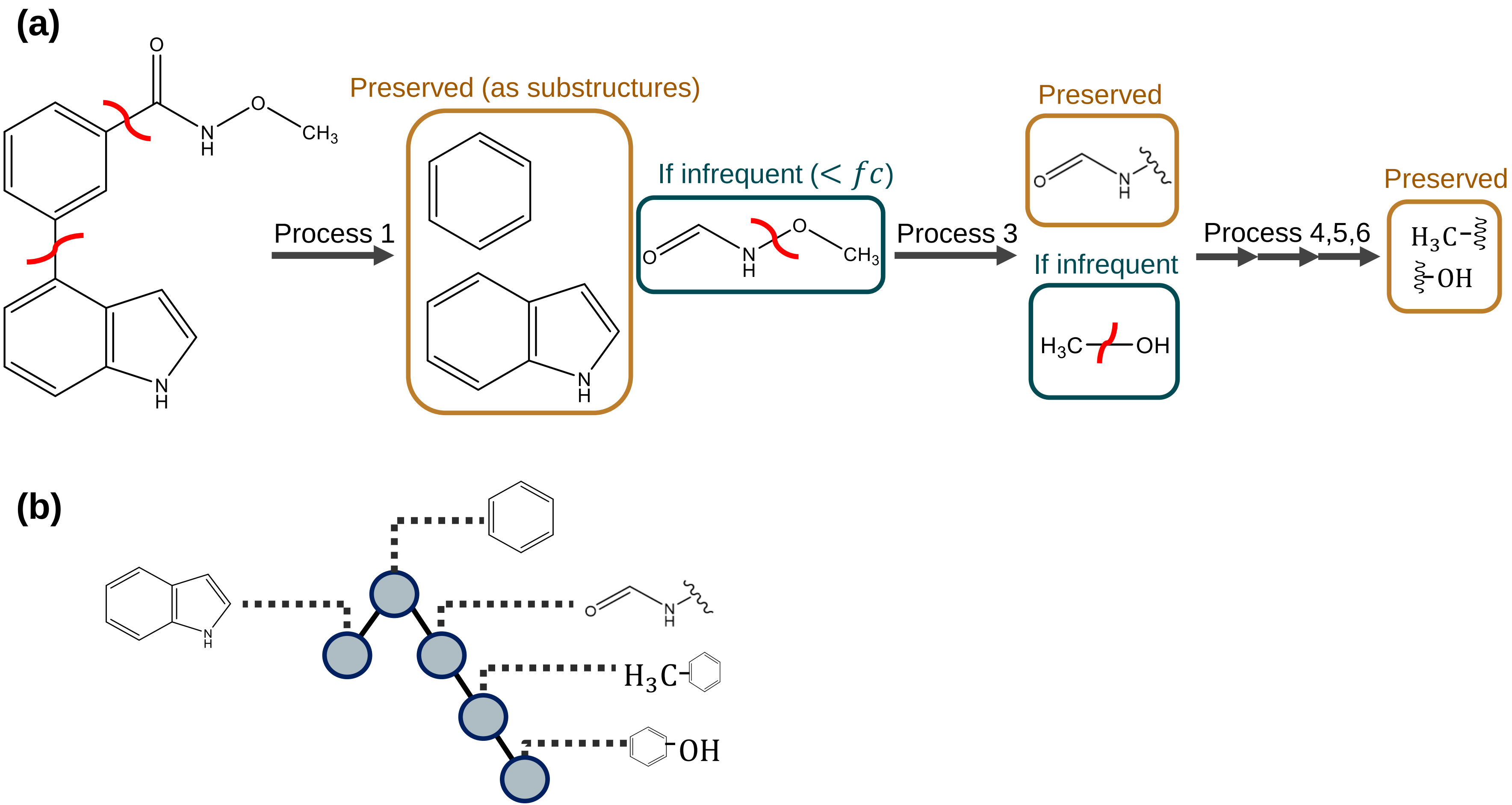

Supplementary Figure S6

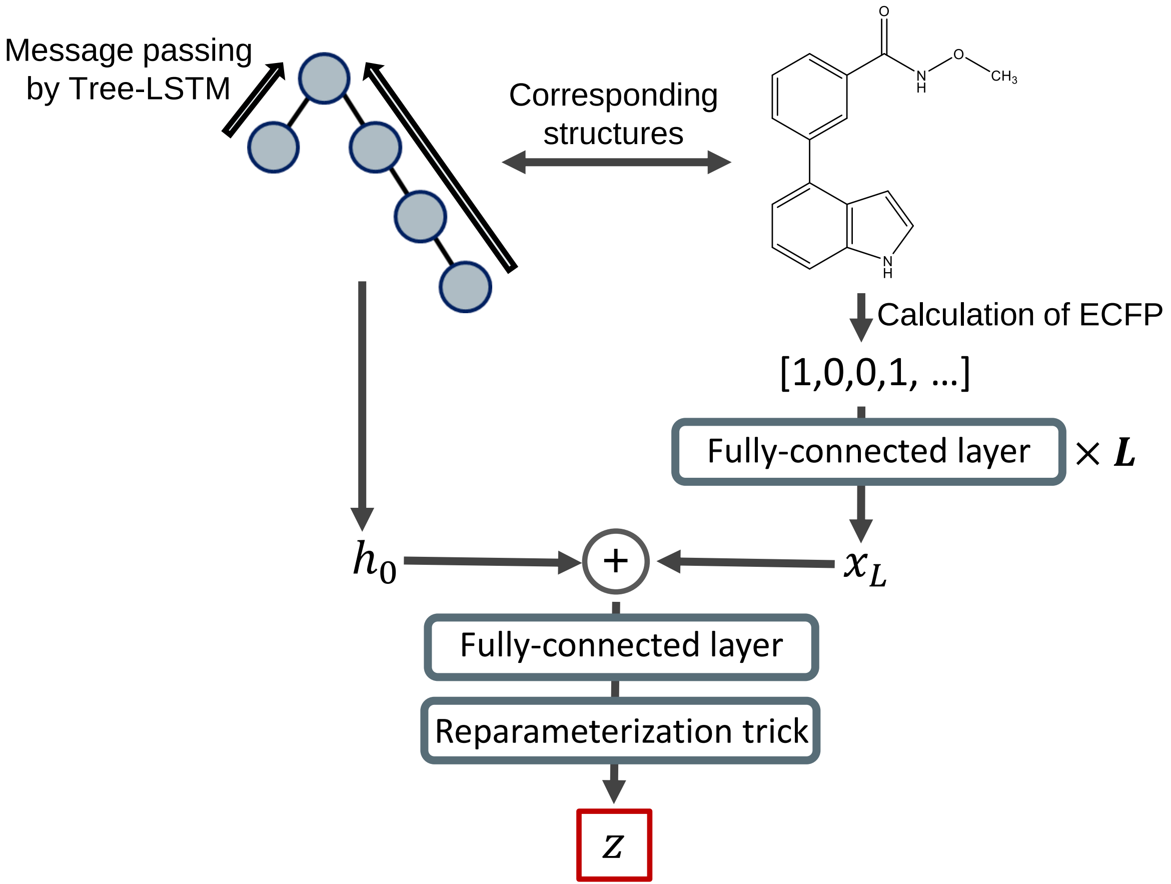

Supplementary Figure S7

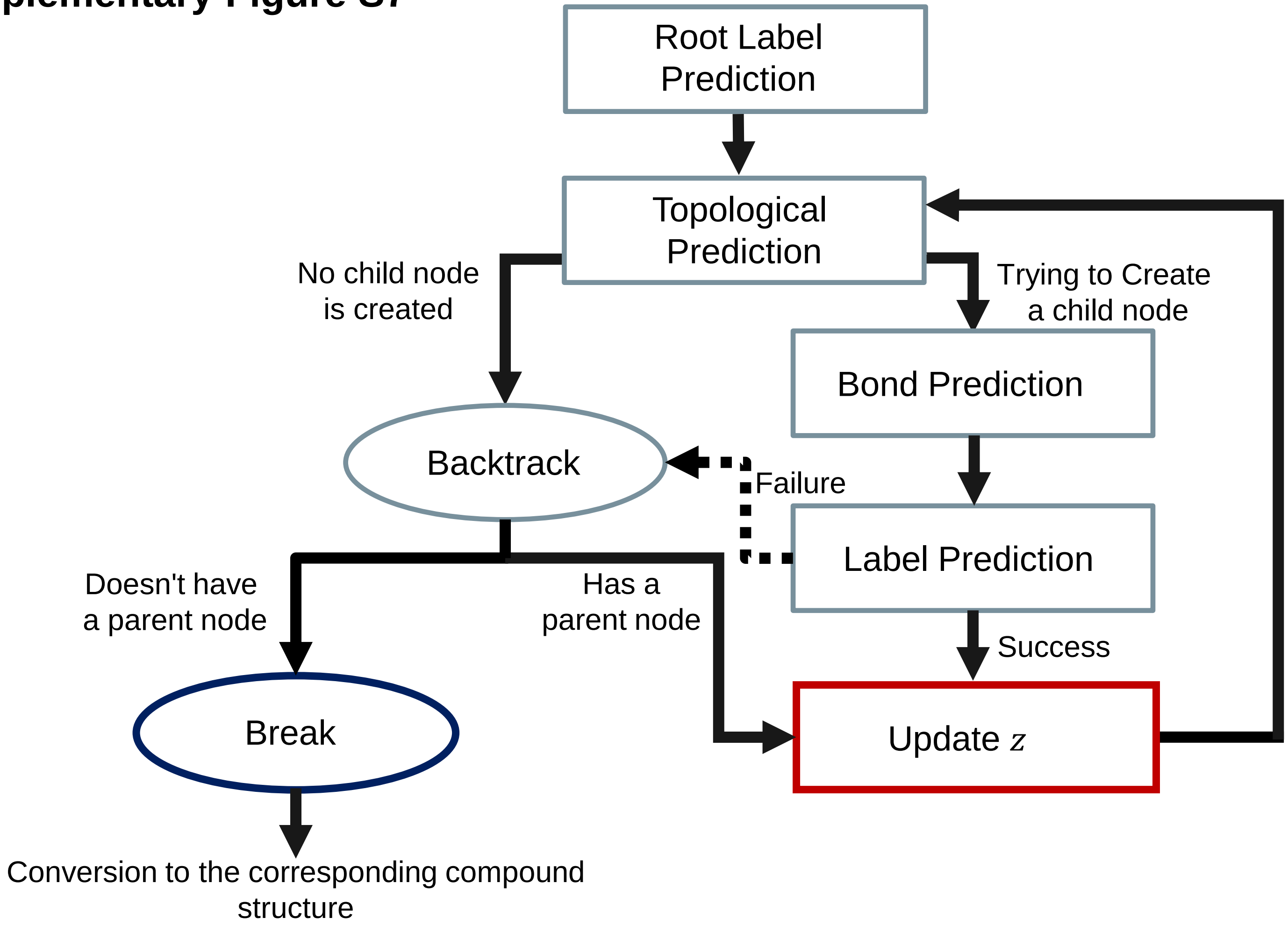

Supplementary Figure S8

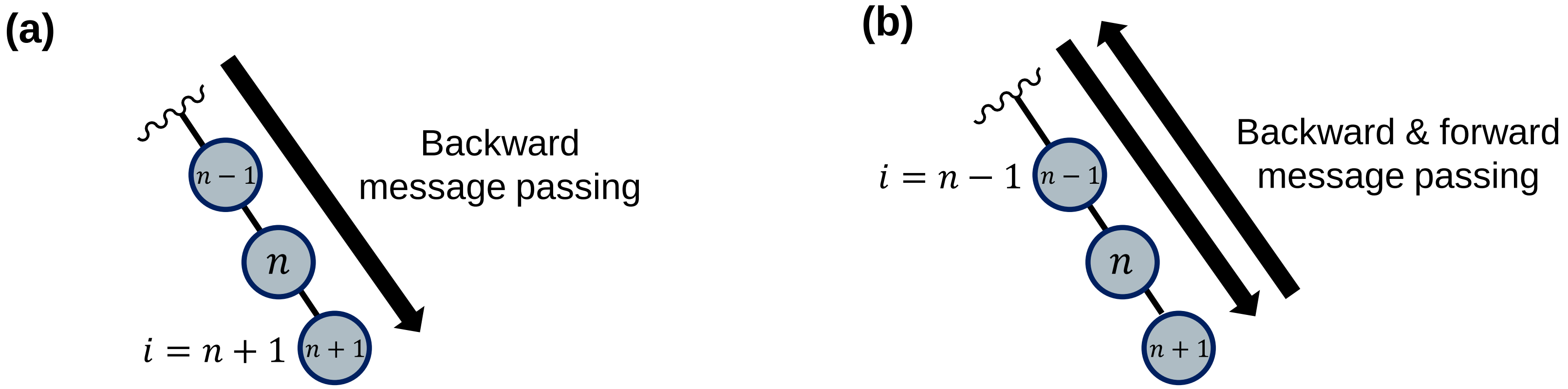

Supplementary Figure S8

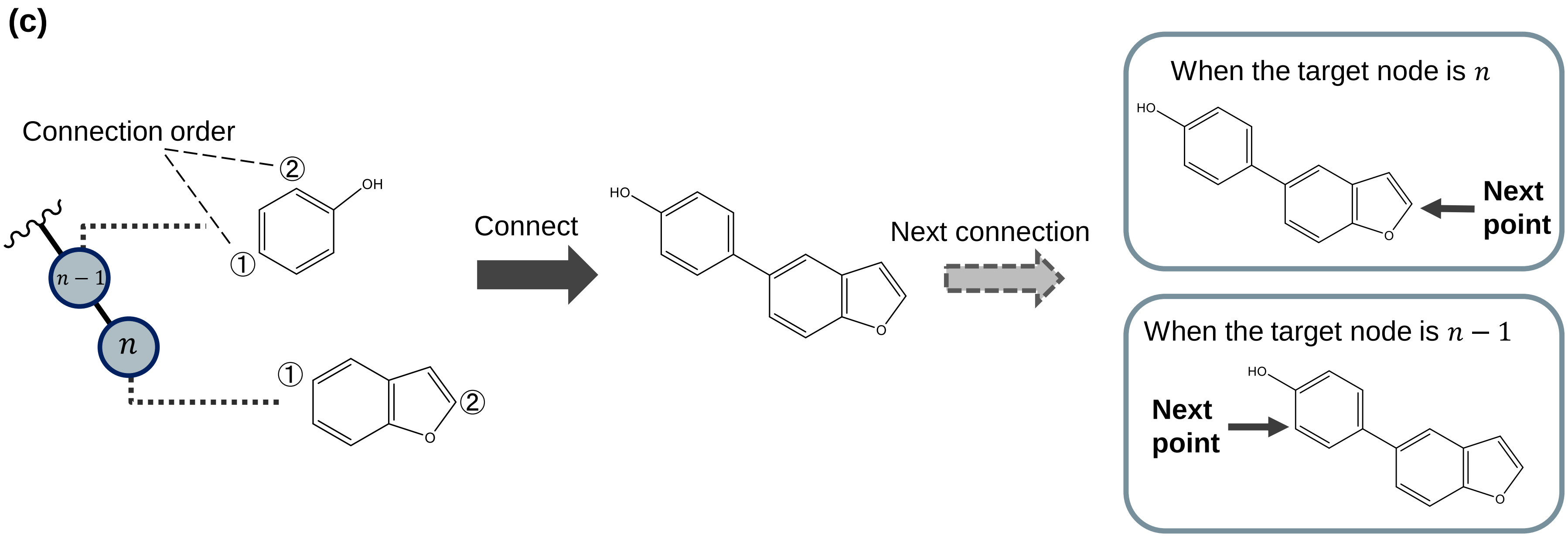

Supplement: Supplementary file 2 — Supplementary material [file 42004_2023_1054_MOESM2_ESM.pdf]
