# Supplementary material for: Variational autoencoder-based chemical latent space for large molecular structures with 3D complexity
Source: Commun Chem. 2023 Nov 16;6:249. doi: 10.1038/s42004-023-01054-6 (PMC10654724; doi:10.1038/s42004-023-01054-6)
Supplement: Supplementary file 3 — Description of Additional Supplementary Files [file 42004_2023_1054_MOESM3_ESM.pdf]

Description of Additional Supplementary Files

File name: Supplementary Data 1

Description: Supplementary data for figures
